# Pregnancy and Tumour: The Parallels and Differences in Regulatory T Cells

**DOI:** 10.3389/fimmu.2022.866937

**Published:** 2022-04-13

**Authors:** Prerana Muralidhara, Vanshika Sood, Vishnu Vinayak Ashok, Kushagra Bansal

**Affiliations:** Molecular Biology and Genetics Unit (MBGU), Jawaharlal Nehru Centre for Advanced Scientific Research (JNCASR), Bangalore, India

**Keywords:** immunological tolerance, maternal-fetal interface, tumour, regulatory T cells (Tregs), immunotherapy

## Abstract

Immunological tolerance plays a critical role during pregnancy as semi-allogeneic fetus must be protected from immune responses during the gestational period. Regulatory T cells (Tregs), a subpopulation of CD4^+^ T cells that express transcription factor Foxp3, are central to the maintenance of immunological tolerance and prevention of autoimmunity. Tregs are also known to accumulate at placenta in uterus during pregnancy, and they confer immunological tolerance at maternal-fetal interface by controlling the immune responses against alloantigens. Thus, uterine Tregs help in maintaining an environment conducive for survival of the fetus during gestation, and low frequency or dysfunction of Tregs is associated with recurrent spontaneous abortions and other pregnancy-related complications such as preeclampsia. Interestingly, there are many parallels in the development of placenta and solid tumours, and the tumour microenvironment is considered to be somewhat similar to that at maternal-fetal interface. Moreover, Tregs play a largely similar role in tumour immunity as they do at placenta- they create a tolerogenic system and suppress the immune responses against the cells within tumour and at maternal-fetal interface. In this review, we discuss the role of Tregs in supporting the proper growth of the embryo during pregnancy. We also highlight the similarities and differences between Tregs at maternal-fetal interface and tumour Tregs, in an attempt to draw a comparison between their roles in these two physiologic and pathologic states.

## Introduction

Our immune system has evolved to protect us from various harmful pathogens such as viruses and bacteria. The immune system achieves this goal by recognizing molecular patterns unique to pathogens, mounting an inflammatory immune response, and eliminating the microorganisms expressing these molecular patterns. An important hallmark of the immune system is its ability to not only distinguish self- and non-self-antigens but also harmful and innocuous foreign antigens- a phenomenon also known as immunological tolerance. A range of ‘central’ and ‘peripheral’ mechanisms render the immune system the ability to maintain the state of immunological tolerance. These include ‘central’ deletion of autoreactive T- and B-cells during development; and active ‘peripheral’ suppression of immuno-reactive T-lymphocytes by a unique population of immunocytes, regulatory T cells (Tregs) (1–3).

Tregs are a subset of CD4^+^ T cells that constitutively express high levels of IL-2 receptor subunit, CD25, on their cell surface and are classically identified as CD4^+^ CD25^+^ T cells (3, 4). The high expression of CD25 on Treg cells allows them to act as ‘IL-2 sink’ and absorb IL-2 from the local microenvironment. This elegant mechanism renders Tregs to inhibit IL-2 dependent proliferation of effector T cells (Teff) and promote their apoptosis (5). A similar IL-2 sequestration-based mechanism has been shown to operate in Treg-mediated regulation of natural killer (NK) cells’ function (6, 7). Another cell surface receptor cytotoxic T lymphocyte antigen 4 (CTLA-4)- that functions as an immune checkpoint- is known to be constitutively expressed on Tregs and has been implicated in Treg-mediated suppression of Teff responses (8, 9). The differentiation, identity, and function of Tregs depend on the expression of lineage-specifying transcription factor Foxp3 (10–13). By virtue of their ability to dampen immune responses, Tregs are not only critical for averting autoimmune diseases, but they also form the cellular basis of resolution of inflammation and tissue repair after the host response to pathogenic infection - a phenomenon also known as immune homeostasis (4, 14–20). Consequently, genetic perturbation of Foxp3 locus in mice leads to loss of Tregs, and these Foxp3-mutant Scurfy mice manifest lethal inflammation and this phenocopies the Foxp3-less disease in humans, immune dysregulation polyendocrinopathy enteropathy X-linked (IPEX) syndrome (13, 21–23).

Evidence gathered over the last two decades has highlighted the role of Tregs as an important negative regulator of immune responses in diverse physiological as well as pathological settings. Pregnancy is one such biological process where Tregs have been implicated to play a crucial and interesting role. During the course of pregnancy, the fetal trophoblast cells emanating from the growing embryo invade the uterine tissue and facilitate the formation of placenta. The growing embryo is a semi-allogenic entity as it derives half of its genetic information from the mother while the other half from the father leading to the expression of antigens that are both foreign as well as self to the mother. A successful pregnancy necessitates that a semi-allogenic fetus is tolerated by the maternal immune system and Tregs actively contribute to the establishment of maternal immune tolerance towards the developing fetus (24–26).

While pregnancy is a physiological phenomenon, tumour is a pathological mimic in terms of tissue invasion. Tumours harbour Tregs that facilitate their survival, and increased tumour Tregs are often associated with a poor prognosis in many cancer types (27–29). The similarities between the placental and tumour microenvironment provide an exciting avenue to understand the phenomenon of local immunosuppression. Understanding the differences between the physiological uterine Tregs and pathophysiological tumour Tregs can provide insights into the development of novel therapies specifically targeted towards tumour Tregs. In this review, we first survey the existing literature on Tregs in pregnancy and cancer. We then attempt to highlight the similarities and differences between Tregs from these two physiologic and pathologic states. This information will serve as a paradigm towards novel immunotherapy-based treatment measures for cancer during pregnancy, and we discuss the challenges and scope of targeting Tregs for treating cancer during pregnancy in the last section of this review.

## Uterine Tregs

The maternal decidua originating from the endometrial lining of the uterus and fetal placenta derived from the trophectoderm of the blastocyst constitute the maternal-fetal interface (30). Interestingly, an extraordinarily large proportion (~40%) of the maternal decidua is composed of immune cells, and T cells (CD3^+^TCRαβ^+^) constitute ~10-20% of maternal leukocytes in the first trimester decidua (30–32). The T cell pool at maternal-fetal interface has cellular repertoire that can have a negative impact on the pregnancy [T helper type 1 (Th1) cells, Th17 cells, cytotoxic T-lymphocytes (CTLs)] as well as cells that can positively influence the fetal growth (Tregs) (33). Hence, a dynamic equilibrium of effector and tolerance compartments of the T cell repertoire is essential to ensure successful placentation and a healthy pregnancy.

On contrary to the general notion that maternal immune responses are in suppressed state at maternal-fetal interface, it was observed that the maternal effector T cells display the potential to be primed by fetal alloantigens and become activated (26, 34, 35). However, this does not result in loss of fetus as tolerance to fetal alloantigens is induced and sustained during pregnancy (34). These observations point towards the establishment of temporal immune tolerance at maternal-fetal interface during pregnancy that licences fetal cells to paradoxically exist in the presence of maternal immune aggression and Tregs play a key role in establishing this tolerance.

The major event that commences the immune activities at maternal-fetal interface is the contact of male seminal fluid with uterine tissue after conception. This leads to infiltration of innate immune cells like dendritic cells (DCs) that traffic paternal antigens to the draining lymph nodes in order to expand the population of thymic and peripheral Tregs that are further recruited to endometrium (36–38). Additionally, male seminal fluid contains various factors like transforming growth factor (TGF)-β, prostaglandin E, and soluble CD38 that can skew T cell fate commitment towards Tregs (39, 40).

Expansion of CD4^+^CD25^+^ Tregs was observed during pregnancy in both mice (25) and humans (31, 41). During the first and second trimester, decidual Tregs constitute a significant proportion (10-30%) of the CD4^+^ T cells (25, 31), and decline postpartum (41). Interestingly, this increase in Treg proportion was not restricted to maternal-fetal interface and expansion of Tregs was also observed in other peripheral tissues of pregnant females (25). These observations suggest that the maternal immune system undergoes a systemic change during the period of gestation. Uterine CD4^+^CD25^+^ T cells expressed Foxp3 messenger RNA confirming their identity as bonafide Tregs (25, 41). Maternal Tregs were also shown to suppress an aggressive allogeneic response directed against the fetus, and their absence led to immunological rejection of the fetus (25). It was also seen that Tregs from both pregnant and non-pregnant mice were able to infiltrate the decidua and placenta of an abortion-prone mice model; however, Tregs only from pregnant mice were capable of preventing fetal rejection *in vivo* (42). These results suggest that Tregs exposed to paternal alloantigens have unique immunoregulatory properties not shared with Tregs from non-pregnant mice. On a similar line, another study demonstrated that frequencies of Tregs increase more in allogeneically pregnant mice compared to syngeneically pregnant mice and these cells contribute to a lowered alloreactivity against paternal antigens (43).

Studies have demonstrated that depletion of Tregs either with anti-CD25 antibodies or using *Foxp3-Dtr* mice promotes maternal-fetal conflicts in allogeneic pregnant mice, but not in syngeneic pregnant mice (44, 45). It has also been observed that loss of Tregs or their dysfunction is associated with several pregnancy-associated disorders such as recurrent pregnancy loss (46) and preeclampsia (47, 48), further emphasizing on the importance of Tregs in immune escape by the growing embryo.

**Figure 1:**
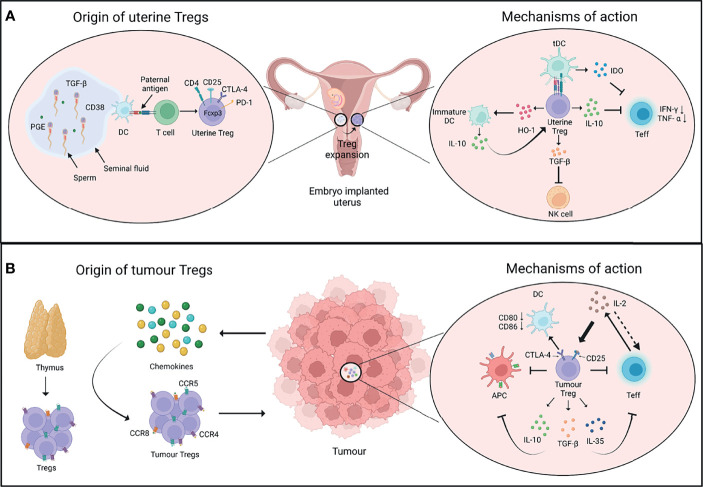
Origin and mechanisms of Tregs in pregnancy and cancer. **(A)** After conception, the seminal fluid encounters the uterine tissue. The presentation of paternal antigens by dendritic cells (DCs) to T cells as well as other soluble factors in the seminal fluid favour the induction of regulatory T cells (Tregs). The uterine Tregs express markers such as CTLA-4, PD-1, and Foxp3, and expand locally in the endometrium during early pregnancy (left panel). Uterine Tregs express factors such as IL-10 which inhibits the proliferation and function of T effector cells (Teff); TGF-β which inhibits the function of cytotoxic natural killer (NK) cells; and HO-1 which maintains decidual DCs in an immature state. These immature DCs express higher levels of IL-10 that further supports the immune-suppressive phenotype of uterine Tregs. Tregs also induce the expression of IDO by tolerogenic DCs (tDCs) which inhibits Teff cell function (right panel). **(B)** Cells in tumour microenvironment release chemokines that recruit Tregs expressing chemokine receptors such as CCR4, CCR5 and CCR8. Tumour Tregs have thymic origin and interact with diverse cell types in the tumour microenvironment (left panel). IL-2 is produced by Teff cells, which is sequestered by Tregs as they constitutively express IL-2 receptor subunit CD25, thus decreasing the bioavailability of IL-2 to Teff cells and inhibiting their function. Tregs may control DCs’ activity through CTLA-4-CD80/86 axis. Tregs also produce immunosuppressive cytokines such as IL-10, TGF-β, and IL-35 that further inhibit the function of Teff cells and antigen-presenting cells (APCs) (right panel). PGE, Prostaglandin E; TGF-β, Transforming growth factor-β; HO-1, Heme oxygenase-1; IDO, Indoleamine 2,3-dioxygenase. Created with BioRender.com.

Tregs exert a range of immune-suppressive, anti-inflammatory, and vascular remodelling functions to support successful embryo implantation in decidua (33, 49). Uterine Tregs exhibit classical attributes of suppressive T cells like elevated expression of CD25, CTLA-4, IL-10, and TGF-β, and prevent effector T cell responses to fetal alloantigens (45, 50–52). Tregs also have an important role in protection from invariant NK T (iNKT) cell-mediated pregnancy loss (50). Tregs also support immune-suppressive phenotype of other cell lineages like macrophages, DCs and uterine NK (uNK) cells to aid in healthy pregnancy (33). And in turn, cross-talk between decidual NK and CD14^+^ myelomonocytic cells initiates a cascade of events promoting Treg induction and immunosuppression (53). In brief, Tregs do not work alone, and both regulate, and are regulated by various other cell lineages, immunomodulatory chemokines, and molecules in ensuring a healthy pregnancy (**Figure 1A**).

## Tumour Tregs

Cancer is viewed as a group of pathological tissue abnormalities that include abnormal cell growth and aberrant gene expression. The progression of cancer is reliant on the interaction between the tumour cells and immunocytes in the surrounding tumour microenvironment, such as tumour infiltrating lymphocytes including Tregs and innate lymphoid cells (ILCs), myeloid-derived suppressor cells (MDSCs), tumour-associated macrophages (TAMs), and tolerogenic dendritic cells (54, 55). Tumour-derived signals often lead to tumour cells evading the immune effector cells, and it has been observed that Tregs are a significant contributor to the immune escape by tumours (56, 57). Thus, Tregs are at crossroads of health and pathology in pregnancy and cancer.

However, on contrary to much believed notion that infiltration of Tregs in tumours results in poor clinical outcomes in various cancers, the role of Tregs in colorectal cancers has been debatable (58, 59). Saito et al. recently demonstrated that colorectal cancers can be categorized into two subclasses based on the degree of infiltration of non-suppressive Foxp3^lo^ T cells that are characterized by absence of naive T cell marker CD45RA and secretion of inflammatory cytokines such as IFN-γ (60). A strong correlation between the frequency of non-suppressive Foxp3^lo^ T cells and the transcription levels of IL-12A and TGF-β1 in colorectal cancer tissues contributed to a better prognosis in colorectal cancer patients. Similar results have been observed in the patients with Hodgkin’s lymphoma, where a high number of Foxp3^+^ cells correlated with longer event free survival, and relapsed samples tended to have a lower frequency of Foxp3^+^ cells (61). These results highlight the phenotypic and functional heterogeneity of Tregs in cancer tissues and warrant a much careful analysis of Treg subtypes in various types of cancers.

Tumour cells or infiltrating innate leukocytes release chemokines such as CCL17 and CCL22 to promote the migration of thymic Tregs expressing receptors such as CCR4, CCR5, and CCR8 from the secondary lymphoid tissues to the site of tumour (62–66). Suppressive nature of Tregs in tumours is supported by the expression of CD25, PD-1 and CTLA-4 on their surface which further shapes cellular architecture of tumours in an immunological sense. For example, high levels of Treg-intrinsic CTLA-4 may aid in suppressing dendritic cells’ activities by affecting CD80 and CD86 expression; while CD25 expression may impact effector T cell and NK cell responses by quenching IL-2 in tumour microenvironment (67, 68). On the other hand, tumour infiltrating myeloid cells such as DCs and MDSCs may support recruitment and differentiation of tumour Tregs *via* secretion of chemokines and cytokines such as TGF-β (69, 70). Overall, an extensive cross-talk of tumour and immune cells with Tregs defines the immunological nature of tumour milieu (**Figure 1B**).

## Uterine *vs.* Tumour Tregs: Close or Poles Apart?

The parallels between maternal-fetal interface and tumour arise from multiple observations- 1) Both fetus and tumour are invasive in nature. 2) These tissues exist in a niche microenvironment. 3) Tregs support the growth of both of these tissues (71). Remarkably, uterine and tumour Tregs also display several similarities in their transcriptional signatures. A recent study by Wienke et al. focused on the transcriptional status of uterine Tregs in myometrial biopsies from maternal-fetal interface and compared the transcriptomic profile of these cells with peripheral blood-derived Tregs and tumour Tregs (72). Uterine Tregs at maternal-fetal interface showed increased suppressive capabilities compared to Tregs in circulation with elevated levels of TIGIT, CD25, IL-10, CTLA-4, OX-40, ICOS, PD-1 and LAG3. This signature is frequently associated with enhanced suppressive capabilities of Tregs or a state also known as “effector Tregs”, especially present in tissues. Not surprisingly, uterine Tregs exhibited features of tissue imprinting with upregulation of genes such as BATF and PRDM1 that are unique to effector Tregs. Interestingly, tumour Tregs also display elevated suppressive activity as well as an effector phenotype with expression of signature genes (66, 73, 74). On similar lines, Wienke et al. observed that the transcriptomic landscape of uterine Tregs displays stronger overlap with that of tumour Tregs, and hepatocellular carcinoma (HCC)-infiltrating Tregs are closest to the uterine Tregs (72, 75). Most importantly, uterine and tumour Tregs-specific gene signature was distant from that of healthy tissue-derived Tregs, suggestive of their unique capabilities (72). These results indicate that both uterine and tumour Tregs acclimatize to the tissue microenvironment, with their major job being active suppression of the local immune responses.

While the similarities between uterine and tumour Tregs have been well understood, there is still scope for further studies on understanding the specific differences between uterine and tumour Tregs. To gather the differences between uterine and tumour Tregs, it is imperative to first acknowledge the dissimilarities between the tissue microenvironment in which these Tregs home. One fundamental difference between maternal-fetal interface and tumour is the inflammatory milieu of the two tissues. Cytokines such as IL-10 in the tumour microenvironment promote a shift towards Th2 responses. Th2 cells support neoplastic growth through limiting CTL activity (76–78) and tumour Tregs may foster Th2 niche in cancer (79). On the other hand, the inflammatory environment during pregnancy is dynamic. It was observed that several pro-inflammatory mediators are required for the chemotaxis of trophoblast cells (80, 81). This pro-inflammatory signature shifts to a dominant Th2 state which is required for the maintenance of pregnancy, while parturition is associated with a shift towards Th1 responses (82, 83). This dynamicity of Th responses highlights the less appreciated heterogeneity of Tregs during the course of gestation and raises the need for a comprehensive study in this direction.

Another major difference is the type of tissue antigens that uterine and tumour Tregs respond to and the origin of these Tregs. During pregnancy, Tregs respond to a mix of ‘maternal self’ and ‘paternal non-self’ antigens. While the initiation of events promoting embryo implantation enables the infiltration of thymic Tregs, it was observed that the decidual environment induces extrathymic expansion of Tregs that are vital for the maintenance of pregnancy (45, 84). On the contrary, it has been suggested that thymic Tregs gain an upper hand in tumour and expand in response to self- and neo-tumour antigens (85, 86). However, there are also reports suggesting tumour conversion of naive T cells into Treg cells, and the question of the origin of tumour Tregs is still of significant interest (87, 88).

**Figure 2:**
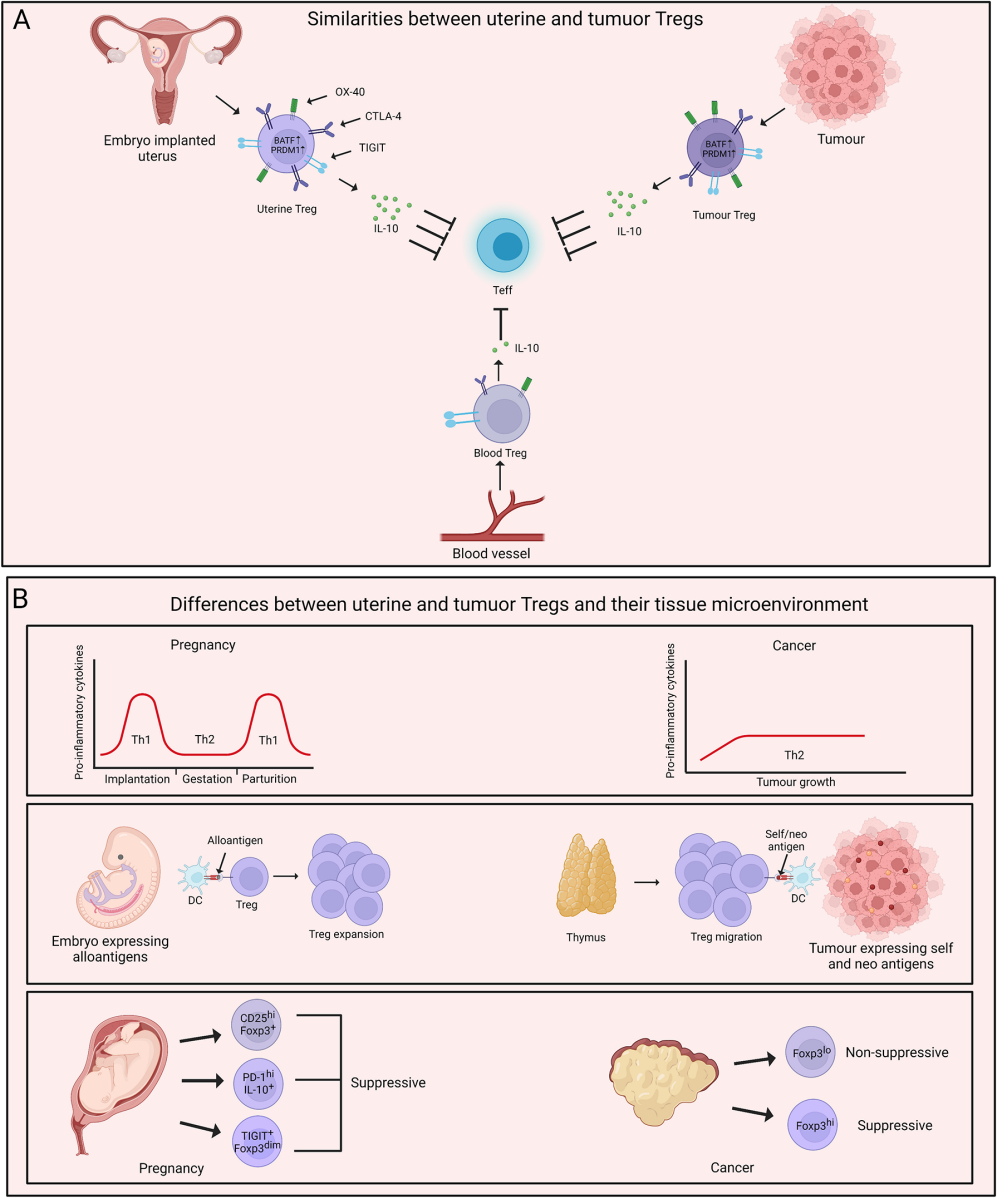
Parallels and dissimilarities between uterine and tumour Tregs. **(A)** Uterine Tregs display several tissue adaptations which mimic tumour Tregs. Both uterine and tumour Tregs are characterised by higher expression of BATF and PRDM1. These cells also demonstrate increased suppressive abilities with elevated levels of IL-10 in comparison to their blood counterparts. Uterine and tumour Tregs exhibit an effector phenotype with increased expression of molecules such as TIGIT, PD-1, OX-40, CTLA-4 and CD25. **(B)** A major difference between uterus and tumour microenvironment is inflammatory milieu of these two tissues. Uterine microenvironment oscillates between T helper type 1 (Th1) and Th2 during pregnancy while tumour growth is fostered by Th2-polarised microenvironment. Uterine Tregs respond to ‘paternal non-self’ and ‘maternal self’ antigens and expand locally. On the contrary, thymus-derived Tregs populate tumour and are exposed to self- and neo-tumour antigens. Uterine and tumour Tregs display phenotypic and functional heterogeneity with uterine Treg subtypes being majorly immune-suppressive while tumour Tregs being both suppressive and non-suppressive. Created with BioRender.com.

The phenotypic and functional heterogeneity of uterine and tumour Tregs adds another layer to their pre-existing complexity. As briefly discussed earlier, two distinct populations of non-suppressive Foxp3^lo^ and suppressive Foxp3^hi^ Tregs home some cancer types such as colorectal cancer and their relative frequency contributes to the disease prognosis (60). Similarly, a recent study by Salvany-Celades et al. highlighted the flavours of Tregs, namely CD25^hi^Foxp3^+^, PD-1^hi^IL-10^+^, and TIGIT^+^Foxp3^dim^ in uterine tissue during pregnancy. These three uterine Treg populations expanded based on different cues offered by diverse cell types at maternal-fetal interface. Interestingly, unlike the Foxp3^lo^ and Foxp3^hi^ cells in colorectal cancer, all three Treg subtypes in the decidua showed suppressive activity on CD4^+^ T cells while only CD25^hi^Foxp3^+^ population reflected consistent suppressive activity on CD8^+^ T cells (84). These observations encourage us to take a step back and have a deeper look at our understanding of uterine and tumour Tregs. **Figure 2** summarizes a head-to-head comparison of uterine *vs* tumour Tregs and their tissue microenvironment.

## Targeting Tregs for Cancer Treatment During Pregnancy: A Double-Edged Sword?

The incidence of cancer during pregnancy is not uncommon. As the mean age for pregnancy is increasing, the potential risk of having malignancies during gestation is also increasing. Pregnancy-associated breast cancer (PABC), melanoma, and other cancer types have been diagnosed in women during pregnancy or in the postpartum period (89–93). Chemotherapy is a viable treatment option for cancer; however, chemotherapy during the first trimester imposes an additional risk of teratogenesis, causing irreversible harm to the fetus. Hence, immune checkpoint inhibitors that target immunosuppressive Tregs can be considered as an alternative therapeutic option for cancer during pregnancy. The most common immune checkpoint inhibitors target PD-1 and CTLA-4 pathways on leukocytes, and both these pathways are crucial for Treg functioning and the maintenance of pregnancy (84, 94–96). Targeting PD-1 and CTLA-4 pathways is also aimed at restoring the function of tumour-infiltrating lymphocytes, which is critical for efficient anti-tumour immunity (97–100). Immunotherapies targeting these immune checkpoint inhibitors can have negative consequences on pregnancy as an altered ratio of immunosuppressive Tregs and pro-inflammatory T cells is associated with pregnancy-related complications (46, 101, 102).

There are few case studies that evaluated the clinical outcome of immunotherapy for cancer during pregnancy; however, the data is dichotomous. It was observed in two independent studies that treatment of melanoma with dual immune checkpoint inhibitors (anti-CTLA-4 and anti-PD-1) did lead to the delivery of healthy babies (103, 104). Similar encouraging observations were made by another recent study and six of seven women that received immunotherapy for melanoma had full-term vaginal deliveries (105). However, a recent review analysed the data from 7 different studies on the therapeutic use of immune checkpoint inhibitors for cancer during pregnancy. This review highlighted complications during pregnancy (71.4%), prematurity (88.9%) and low birth weight (1267g) following immunotherapeutic treatment of cancer (106). These results suggest that immunotherapy for cancer may not always be fatal for fetus but the course of gestation may involve various pregnancy-related complications. Most importantly, the lack of larger cohorts in these studies is a major drawback. Hence, the efficacy and safety of current immunotherapy regimens during pregnancy remains debatable. More extensive studies and scientific discussion is needed before Treg-directed immunotherapies for cancer during pregnancy can become a norm. And, the studies highlighting differences between uterine and tumour Tregs can open new immunotherapy based avenues for the treatment of cancer in pregnant women.

## Conclusions and Future Perspectives

The immunological underpinnings of Tregs at maternal-fetal interface and in tumours have greatly enhanced our understanding of how physiological and pathological processes of pregnancy and cancer ensue. Uterine and tumour Tregs display significant overlap in their transcriptional signatures; however, future studies should focus on dissimilarities between these Treg types. This knowledge will not only help in answering questions on immunotherapies for cancer during pregnancy, but may also contribute to the development of novel immunological treatment regimens for other pregnancy-associated disorders such as preeclampsia and recurrent pregnancy loss. Moreover, larger cohorts will aid in evaluating novel Treg-based immunotherapies for cancer during gestation or in postpartum period.

In conclusion, our understanding of immunological attributes during health and disease has improved tremendously in recent times and this knowledge needs to be harnessed for safer pregnancies and treating diseases like cancer.

## Author Contributions

PM and VS wrote the first draft of the manuscript. VS created the figures. KB and VV reviewed and edited the manuscript. KB supervised the study. All authors contributed to the manuscript and approved the submitted version.

## Funding

Authors thank JNCASR and DBT/Wellcome Trust India Alliance for intramural and extramural financial support respectively. PM acknowledges the scholarship from Council of Scientific & Industrial Research (CSIR), Government of India. VS is supported by research fellowship from JNCASR. VV is supported by funds from DBT/Wellcome Trust India Alliance Intermediate Fellowship (IA/I/19/1/504276). KB is a recipient of DBT/Wellcome Trust India Alliance Intermediate Fellowship (IA/I/19/1/504276).

## Conflict of Interest

The authors declare that the research was conducted in the absence of any commercial or financial relationships that could be construed as a potential conflict of interest.

## Publisher’s Note

All claims expressed in this article are solely those of the authors and do not necessarily represent those of their affiliated organizations, or those of the publisher, the editors and the reviewers. Any product that may be evaluated in this article, or claim that may be made by its manufacturer, is not guaranteed or endorsed by the publisher.
